# Supplementary material for: Dynalign II: common secondary structure prediction for RNA homologs with domain insertions
Source: Nucleic Acids Res. 2014 Nov 21;42(22):13939–48. doi: 10.1093/nar/gku1172 (PMC4267632; doi:10.1093/nar/gku1172)
Supplement: SUPPLEMENTARY DATA [file supp_gku1172_nar-02021-z-2014-File012.zip › manual/javadoc/RNAstructure_java_interface/source/menus/package-summary.html]

RNAstructure\_java\_interface.source.menus


JavaScript is disabled on your browser.


- Overview
- Package
- Class
- Tree
- Index
- Help

- Prev Package
- Next Package

- Frames
- No Frames

- All Classes

# Package RNAstructure\_java\_interface.source.menus

A package that holds all menus used in the Java RNAstructure interface.

See: Description

- Class Summary

  | Class | Description |
  | --- | --- |
  |  |  |
  | --- | --- |
  | ConstraintsMenu | A class that creates a folding constraints menu. |
  | DynamicMenuBar | A class that creates a menu bar. |
  | FileMenu | A class that creates a "File" menu. |
  | HelpMenu | A class that creates a "Help" menu. |
  | NucleicAcidMenu | A class that creates a menu which holds commands specific for a nucleic acid type. |
  | RolloverMenu | A class that creates a menu for the RNAstructure GUI. |


## Package RNAstructure\_java\_interface.source.menus Description

A package that holds all menus used in the Java RNAstructure interface.

- Overview
- Package
- Class
- Tree
- Index
- Help

- Prev Package
- Next Package

- Frames
- No Frames

- All Classes
